# Co-occurrence of PTSD and affective symptoms in a large sample with childhood trauma subtypes: A network analysis

**DOI:** 10.3389/fpubh.2023.1093687

**Published:** 2023-03-07

**Authors:** Yu Jin, Shicun Xu, Zhishan Hu, Jiaqi Li, Hui Li, Xiaofeng Wang, Xi Sun, Yuanyuan Wang

**Affiliations:** ^1^College of Education for the Future, Beijing Normal University, Beijing, China; ^2^Northeast Asian Research Center, Jilin University, Changchun, China; ^3^Department of Population, Resources and Environment, Northeast Asian Studies College, Jilin University, Changchun, China; ^4^China Center for Aging Studies and Social-Economic Development, Jilin University, Changchun, China; ^5^Shanghai Mental Health Center, Shanghai Jiao Tong University School of Medicine, Shanghai, China; ^6^Key Laboratory of Brain, Cognition and Education Sciences, Ministry of Education, China; School of Psychology, Center for Studies of Psychological Application, and Guangdong Key Laboratory of Mental Health and Cognitive Science, South China Normal University, Guangzhou, China; ^7^School of Public Health, Jilin University, Changchun, China

**Keywords:** childhood trauma, psychopathological symptoms, college students, network analysis, anxiety, depression, PTSD

## Abstract

**Background:**

Exposure to childhood trauma (CT) is associated with various deleterious mental health outcomes, increasing the risk of suicidal behaviors. The objective of this study is to investigate the different effects of three forms of CT, including emotional abuse (EA), physical abuse (PA), and sexual abuse (SA), on potential psychopathological symptoms among college students.

**Methods:**

A total of 117,769 students from 63 Chinese colleges participated in this study. There were 1,191 participants in the EA group (1.24%; 95% CI: 1.17–1.31%), 1,272 participants in the PA group (1.32%; 95% CI: 1.25–1.40%), and 3,479 participants in the SA group (3.62%; 95% CI: 3.50–3.73%). CT was measured by the Childhood Trauma Questionnaire-Short Form. Psychopathological symptoms (i.e., depression, anxiety, and PTSD) were measured by the PHQ-9, GAD-7, and Trauma Screening Questionnaire, respectively. Network analysis was applied to analyze psychopathological symptoms between three CT subgroups (EA, PA, and SA). The associations and centralities of the networks were calculated, and the network characteristics of the three subgroups were contrasted.

**Results:**

The main symptoms across all three groups are uncontrollable worry, sad mood, irritability, and fatigue, which indicates these core symptoms play essential roles in maintaining the whole psychological symptoms network. Furthermore, there are significant differences in symptom associations between the three groups. The comparison of network structures of the three groups shows that the SA group reports more PTSD symptoms, the EA group reports more suicide-related symptoms, and the PA group reports more anxiety symptoms.

**Conclusion:**

Specific symptoms were disclosed across each group by the distinctive core psychopathological symptoms found in the CT subgroup networks. The present study's findings show different associations between CT and psychopathology and may help classify potential diagnostic processes. Therefore, local governments and academic institutions are recommended for early intervention to promote the psychological well-being of CT survivors.

## Highlights

- Central symptoms within the three subgroups of childhood trauma (EA, PA and SA) are uncontrollable worry, sad mood, irritability, and fatigue;
- The sexual abuse group reports more associations with PTSD symptoms;
- The emotional abuse group reports more associations with suicide-related symptoms;
- The physical abuse group reports more associations with anxiety symptoms.

## Introduction

Childhood trauma (CT) can occur when a child witnesses or experiences overwhelming adverse events in childhood (1, 2). These can happen in relationships that involve abuse, assault, neglect, violence, exploitation, or bullying. Chronic CT causes self-defeating cognition and behaviors that hinder the healthy development of schemata (3). Moreover, previous studies have found that exposure to CT has long-term consequences (4), including depressive disorders, anxiety disorders, posttraumatic stress disorder (PTSD), self-injurious behaviors, and suicidal behavior (5–8). Three varieties of CT are frequently acknowledged: emotional abuse (EA), physical abuse (PA), and sexual abuse (SA) (7, 9–12). Globally, the prevalence of child SA varies from 2 to 62%, while the annual prevalence of PA ranges from 4 to 16%, and around 10% of children experience EA or neglected abuse (13).

Several systematic reviews and meta-analyses have found that those exposed to any form of CT are much more likely to report chronic mental health conditions (5–8). For example, McKay and colleagues reported that those exposed to multiple forms of CT have more than three times the odds of developing a mental disorder (6). Another meta-analysis reported that childhood adversities significantly increased psychosis across all research designs, with an overall effect of increasing 2.78 times (8). In addition, CT may increase the risk of depression, anxiety, PTSD, and so on. However, although many studies investigate the associations between CT and psychopathological symptoms, such as PTSD, depression, and anxiety, the interaction studies between PTSD, depression, and anxiety among individuals exposed to different types of CT are yet to be comprehensively explored.

Network analysis is one approach to describe the dynamics between symptoms from a psychopathology perspective (14). Compared with traditional latent variable models, network analysis could capture and express the correlation and structure of the observed variables (15, 16). According to the network perspective, an episode of disorder occurs whenever the necessary amount of symptoms get active for a while (17). Recovery from an illness happens when symptoms deactivate, the association between them dissolves, or both. Hence, network analysis helps guide therapeutic intervention or treatment for psychological diseases (14). In network analysis, specific symptoms (defined as nodes) are observed variables that may have solid causal links with one another. Moreover, a bridge symptom is a bridge between two other symptoms in the network (15). Similarly, specific associations (defined as edges) between two symptoms may be substantially more robust and influential than other edges (15). In a network structure, a vital edge may be thought of as a strong association between two symptoms that are likely to occur or non-occur at the same time (18). To date, the network analysis contributed to advance psychopathology research (17, 19–24). For instance, Mullarkey et al. reported that self-hatred, loneliness, and sadness are the most central symptoms of adolescent depression (23), suggesting that these symptoms (and relationships between symptoms) should be prioritized in theoretical frameworks for teenage depression and may also serve as significant therapeutic targets for adolescent depression therapies. In addition, Network analysis gives hints to the causal mechanism behind PTSD in those who have experienced a severe natural catastrophe (19) and the association between PTSD symptoms in the acute phase after trauma and the chronic phase (20). Moreover, Bryant et al. used network analysis to simulate the development of PTSD symptoms over time and to pinpoint the most crucial target symptoms for early intervention (20). McNally et al. employed it to illustrate how major depressive illness and obsessive-compulsive disorder are symptomatically related (22).

Therefore, the current study analyzed psychopathological symptoms using network analysis, which aimed to go beyond the level of symptom severity and to understand which symptoms might be particularly central to the experience of CT. We used network approaches to identify the strong association between symptoms among individuals exposed to different types of CT. Furthermore, significant differences in network structures among various CT groups could be found, which could provide advice on intervention or treatment to decrease the risk of psychosis for specific CT groups. The objectives of this study were to examine the associations between psychopathological symptoms, including PTSD, depression, and anxiety, across three subgroups (individuals exposed to EA, PA, and SA). Additionally, to identify central symptoms in the network structure within the three CT groups. Finally, to compare the three groups to find the significant differences in network interactions.

## Methods

### Study design and settings

This study was part of a large-scale, cross-sectional study covering all the college students in Jilin province, China. The data was collected between 26 October and 18 November 2021. Sixty-three colleges were invited to this study. The introduction, invitation to participate in this study and access to the questionnaire were all connected using a Quick Response code (QR Code), which was created and implemented. All the colleges in Jilin province distributed the QR code and then forwarded it to students.

The inclusion criteria for the current study included: (1) aged 16 years or older; (2) students studying in universities or colleges in Jilin province, China; (3) able to understand the assessment content and Chinese Ethical approval for this study was granted by Jilin University. In addition, all participants provided electronic informed consent.

### Measurements

#### Childhood trauma questionnaire-short form

Childhood trauma was measured by the Childhood Trauma Questionnaire-Short Form (CTQ-SF), which was developed by Bernstein et al. (25) and has been examined the validity and reliability in Chinese version (26). The CTQ-SF contains 28 items, a self-report inventory developed to measure five types of abuse or neglect in childhood or adolescence. It evaluates five domains of childhood abuse and neglect: sexual abuse (SA), physical abuse (PA), emotional abuse (EA), physical neglect (PN), and emotional neglect (EN). Participants are queried on items with a 5-point, Likert-type answer format ranging from “1” (never true) to “5” (very often true). Each subscale (i.e., SA, PA, EA, PN, and EN) contains five items, and additional three items are intended to measure any tendency to minimize or deny the abuse (MD subscale). Moderate-severe cutoff scores for each subscale are ≥13 for EA, ≥10 for PA, and ≥8 for SA.

#### Patient Health Questionnaire-9

Depressive symptoms were measured by the Chinese version of Patient Health Questionnaire-9 (PHQ-9) (27), which has been well-validated in the Chinese population (28). The PHQ-9 consists of nine items covering cognitive, emotional, physiological, and interpersonal symptoms of depression. The items of PHQ-9 and their reference names are listed in Supplementary Table 1. Each item was given a score ranging from “0” (not at all) to “3” (nearly every day), with a higher score indicating more severe depression symptoms. The sum score of PHQ-9 was used to measure participants' depression (Cronbach's alpha = 0.915).

#### Generalized anxiety disorder scale-7

Anxiety symptoms were assessed by Generalized Anxiety Disorder Scale-7 (GAD-7) (29), which comprises seven items, with each scored scoring from “0” (not at all) to “3” (nearly every day), with higher scores indicating more severe anxiety symptoms. This instrument asks participants about the frequency of a series of anxiety symptoms in the past 2 weeks (see Supplementary Table 1). The GAD-7 has been well-validated in the Chinese population (30). Moreover, the sum score of the GAD-7 was used to measure participants' anxiety (Cronbach's alpha = 0.928).

#### Trauma Screening Questionnaire

To identify the post-traumatic stress disorder (PTSD) symptoms, the Trauma Screening Questionnaire (TSQ) was adopted in this study. TSQ is a 10-item self-report scale with a yes/no response format. It assesses the presence of 5 intrusion items (e.g., “Upsetting dreams about the event”) and five hyperarousal items (e.g., “Difficulty falling or staying asleep”) over the past week (31). It was adapted from the PTSD Symptom Scale-Self-Report Version (PSS-SR) (32). The TSQ is a validated screening tool for PTSD with a sensitivity and specificity of 0.85 and 0.89, respectively (31). In addition, this scale has been validated in the Chinese population (33). The reference names of ten items are listed in Supplementary Table 1.

### Statistical analysis

#### Network estimation

Partial correlation networks were used to assess the association between depression, anxiety, and PTSD symptoms among participants with EA, PA, and SA. The Graphical Gaussian Model (GGM) with the graphic least absolute shrinkage and selection operator (LASSO) was used to build the network model. Extended Bayesian Information Criterion (EBIC) was performed for parameter selection. For each node, expected influence (EI) represents the summed weight of all its edges, positive and negative, with its immediate neighbor nodes in the network (35). Note that the sign of the association connecting two nodes (i.e., negative vs. positive partial correlation) was considered when calculating the EI. All the analyses used the R package “qgraph” (15, 36). The accuracy and stability of the network model would be assessed by confidence intervals (CIs) (37), the correlation stability coefficient (CS-C) (15, 38) and the bootstrapped difference tests (18).

#### Network comparison

To examine statistical differences among the networks of three different abuse groups, the Network Comparison Test (NCT) was performed. This methodology assesses the difference in network structure (e.g., distributions of edge weights), global strength (e.g., the absolute sum of all edge weights of the networks), and each edge between the two networks (i.e., participants with EA vs. those with PA; participants with EA vs. those with SA; participants with PA vs. those with SA) using Holm-Bonferroni correction of *p*-values due to multiple tests (35). The significant edge differences for each pair of groups were plotted after these testing. These tests were performed with the R-package “NetworkComparisonTest” version 2.0.1 (34).

## Results

### Study sample

A total of 117,769 students were invited to participate, while 96,218 fulfilled the study entry criteria and completed the assessment (40,065 males and 56,153 females). Among the whole 96,218 participants, there are 1,191 participants in the EA group (1.24%; 95% CI: 1.17–1.31%), 1,272 participants in the PA group (1.32%; 95% CI: 1.25–1.40%), and 3,479 participants in the SA group (3.62%; 95% CI: 3.50–3.73%). The participants' characteristics are listed in Table 1. Most participants are undergraduate students, mean age (of 19.59 ± 1.74), and 58.4% are females. There are significant differences between the three CT groups among the mean scores of PHQ-9, GAD-7, and TSQ-10 (all *P* < 0.001). As in Table 1, the EA group has the worst PHQ-9, GAD-7, and TSQ-10. Furthermore, there are significant differences between the three groups in sex proportion and family type (*P* < 0.001).

**Table 1:** Participants' sociodemographic characteristics.

	**Emotional abuse (*N* = 1,191)**	**Physical abuse (*N* = 1,272)**	**Sexual abuse (*N* = 3,479)**	***X*^2^/*F***	***P*-value**
Females (%)	856 (71.9)	528 (41.5)	1,780 (51.2)	242.4	**< 0.001**
Education level *N* (%)				4.4	0.63
First year	558 (46.9)	608 (47.8)	1,626 (46.7)		
Second year	323 (27.1)	356 (28.0)	915 (26.3)		
Third year	181 (15.2)	183 (14.4)	566 (16.3)		
Fourth year	129 (10.8)	125 (9.8)	372 (10.7)		
Residence				13.0	0.002
City	673 (56.5)	709 (55.7)	1,788 (51.3)		
Town and county	518 (43.5)	563 (44.3)	1,691 (48.6)		
Ethnic				1.5	0.48
Han	1,057 (88.7)	1,127 (88.6)	3,119 (89.7)		
Others	134 (11.3)	145 (11.4)	360 (10.3)		
Family type				141.5	**< 0.001**
Nuclear family	644 (54.1)	798 (62.7)	2,272 (65.3)		
More than three generation	173 (14.5)	238 (18.7)	651 (18.7)		
Adoptive/foster	6 (0.5)	1 (0.1)	6 (0.2)		
Reconstituted	91 (7.6)	59 (4.6)	119 (3.4)		
Single-parent	223 (18.7)	149 (11.7)	357 (10.3)		
Left-behind	54 (4.5)	27 (2.1)	74 (2.1)		
Current annual income, N (%)				15.3	0.02
< ¥6,000	375 (31.5)	373 (29.3)	1,052 (30.2)		
¥6,000–13,999	406 (34.1)	395 (31.1)	1,047 (30.1)		
¥14,000–22,999	168 (14.1)	224 (17.6)	553 (15.9)		
≥¥23,000	242 (20.3)	280 (22.0)	827 (23.8)		
Only child				0.2	0.89
Yes	572 (48.0)	620 (48.7)	1,698 (48.8)		
No	619 (52.0)	652 (51.3)	1,781 (51.2)		
				F	
Age, years: mean (SD)	18.98 (4.26)	19.51 (3.92)	19.67 (4.05)	6.4	0.06
PHQ-9	11.25 (6.35)	7.92 (5.19)	6.91 (4.92)	299.1	**< 0.001**
GAD-7	8.54 (5.62)	5.74 (4.56)	5.18 (4.34)	231.4	**< 0.001**
TSQ-10	6.10 (2.91)	4.45 (3.05)	4.32 (3.14)	153.3	**< 0.001**

PHQ-9: Patient Health Questionnaire-9; GAD-7: Generalized Anxiety Disorder Scale-7; TSQ-10: Trauma Screening Questionnaire-10; SD: standard deviation. Categorical variables were compared by the Chi-squared test. Continuous variables were compared by One-way analysis of variance (ANOVA). Bold values refer to existing the significant difference with *P* < 0.001.

### Network estimation and centrality measure results

**Figure 1:**
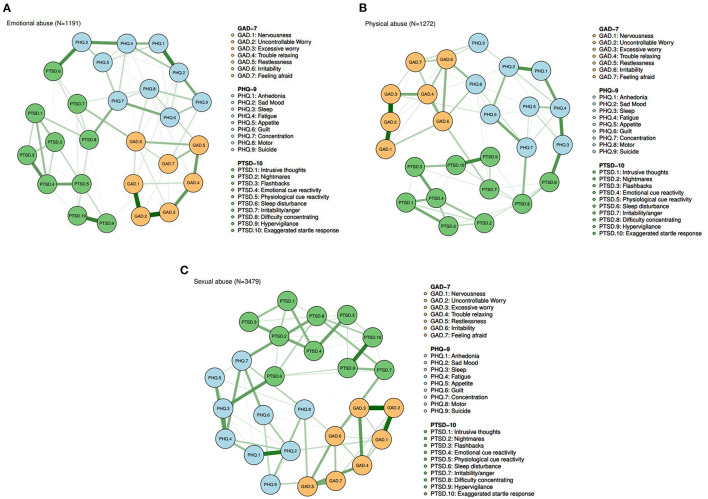
**(A)** Symptom network among participants with emotional abuse (*N* = 1,191). **(B)** Symptom network among participants with physical abuse (*N* = 1,272). **(C)** Symptom network among participants with sexual abuse (*N* = 3,479).

The network estimation among the three groups is displayed in Figures 1A–C. The results show that depressive, anxiety, and PTSD symptoms were positively correlated with each other. Moreover, the network models were similar across the three groups in terms of the strongest edges, including GAD.2 (“Uncontrollable worry”)—GAD.3 (“Excessive worry”), GAD.1 (“Nervousness”) —GAD.2 (“Uncontrollable worry”), PTSD.9 (“Hypervigilance”) —PTSD.10 (“Exaggerated startle response”), and PHQ.1 (“Anhedonia”) —PHQ.2 (“Sad mood”).

**Figure 2:**
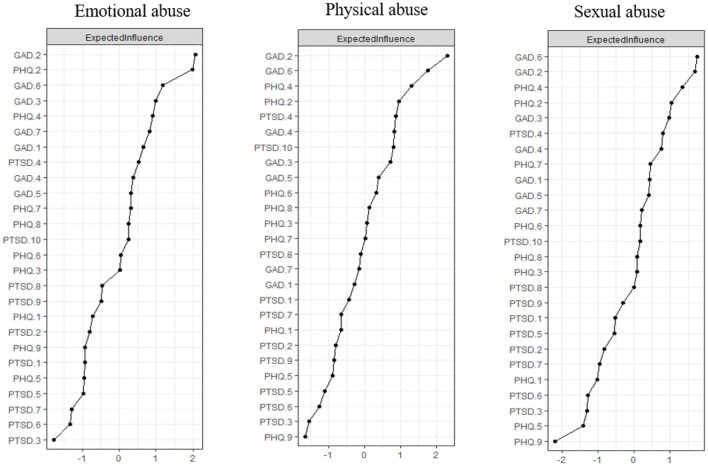
Expected influence of each node in the networks among participants with emotional abuse, physical abuse and sexual abuse.

Figure 2 shows the EI of each node in the networks among participants with EA, PA, and SA. The strength, betweenness, and closeness of each node among the three groups are displayed in Supplementary Figure 1. For participants with EA, nodes GAD.2 (“Uncontrollable worry”), PHQ.2 (“Sad mood”), and GAD.6 (“Irritability”) had the highest EI centrality. For participants with PA, the nodes with the three highest EI were GAD.2 (“Uncontrollable worry”), GAD.6 (“Irritability”), and PHQ.4 (“Fatigue”). For participants with SA, GAD.6 (“Irritability”), GAD.2 (“Uncontrollable worry”), and PHQ.4 (“Fatigue”) had the highest EI centrality, which was similar to the PA group.

### Network accuracy and centrality

The analysis of the accuracy of edges, as implemented utilizing non-parametric CIs, revealed that the precision of edges was acceptable, with smaller CIs indicating a more accurate estimation of edges (Supplementary Figure 2). Additionally, the bootstrapped difference tests showed that many comparisons between edge weights were statistically significant (Supplementary Figure 3). The case-dropping subset bootstrap approach demonstrated that even after eliminating substantial chunks of the sample, the betweenness, closeness, and strength values remained consistent (Supplementary Figures 4, 5). EI and Strength showed an excellent level of stability (CS-C = 0.75) among the three groups, while the levels of stability for betweenness (CS-C ≈ 0.51) and closeness (CS-C ≈ 0.28) were less than optimal.

### Network comparison results

**Figure 3:**
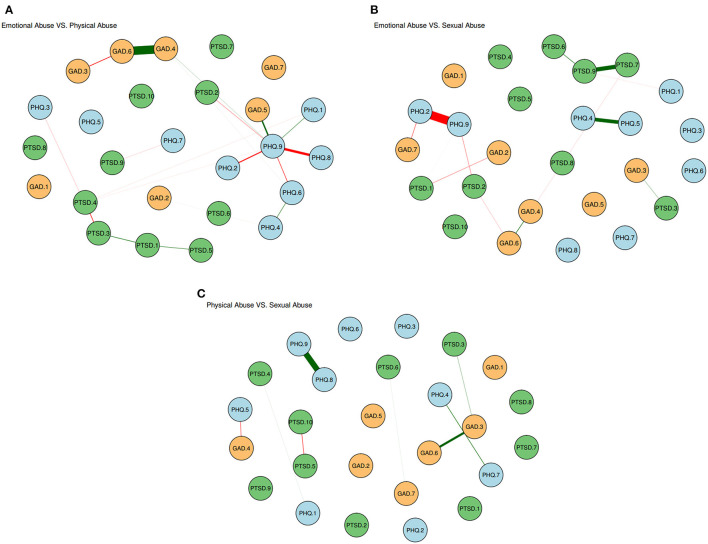
**(A)** Edge significant differences between participants with emotional abuse and physical abuse. The red edges denote the stronger associations in emotional abuse group while the green edges denote the stronger associations in physical abuse group. **(B)** Edge significant differences between participants with emotional abuse and sexual abuse. The red edges denote the stronger associations in emotional abuse group while the green edges denote the stronger associations in sexual abuse group. **(C)** Edge significant differences between participants with physical abuse and sexual abuse. The red edges denote the stronger associations in physical abuse group while the green edges denote the stronger associations in the sexual abuse group.

A comparison of network models among the three groups was conducted. There were significant differences between EA and PA groups in edge weights (*p* = 0.015) and between EA and SA groups in network global strength (*p* = 0.019) (Supplementary Figures 1–3). Figures 3A–C show significant differences in edge weights among the three groups. The significant differences among the three groups would be described as follow.

Compared with EA and PA groups, the SA group is significantly more likely to present symptoms associated with PTSD symptoms, such as PTSD.7 (“Irritability”)—PTSD.9 (“Hypervigilance”), PTSD.6 (“Sleep disturbance”)—PTSD.9 (“Hypervigilance”), and PTSD.3 (“Flashbacks”)—PTSD.4 (“Emotional cue activity”). Compared with PA and SA groups, the EA group is significantly more likely to present symptoms associated with symptom PHQ.9 (“Suicide”), such as PHQ.9 (“Suicide”)—PHQ.2 (“Sad mood”), PHQ.9 (“Suicide”)—PHQ.8 (“Motor”). Compared with EA and SA groups, the PA group is significantly more likely to present symptoms associated with anxiety symptoms, such as GDA.4 (“Trouble relaxing”)—GAD.6 (“Irritability”), and GAD.5 (“Restlessness”)—PHQ.9 (“Suicide”).

## Discussion

This is a large-scale network analysis to investigate the interaction of PTSD, depression, and anxiety symptoms among college students exposed to three types of CT (EA, PA, and SA). The highest prevalence was SA group, with 3.62% among participants. The results showed that central symptoms within the three groups are uncontrollable worry, sad mood, irritability, and fatigue, which indicates these core symptoms play essential roles in maintaining the whole psychological symptoms network. Results also revealed significant differences across three groups: the SA group is more likely to be associated with PTSD symptoms, the EA group is more likely to be associated with suicide-related symptoms, and the PA group is more likely to be associated with anxiety symptoms.

The current study presented several remarkable advantages. Although research has successfully established a connection between CT and psychopathological symptoms (5–7), the potential associations that may account for the connection are still not fully understood. This study revealed associations between psychological symptoms among college students exposed to different types of CT. Central symptoms of the network structures, which integrated depression, anxiety, and PTSD symptoms, were almost the same among the three subgroups. Our result showed that uncontrollable worry, sad mood, irritability, and fatigue might trigger other symptoms in psychological symptoms dynamics when college students are exposed to EA, PA, or SA. This result would be helpful to therapeutic intervention or future treatment for psychological diseases.

Consistent with previous systematic reviews and meta-analyses, the current study also found that CT experiences increase the risk of depression and anxiety (6, 7, 39, 40). Our results align with the existence of an affective pathway to psychological symptoms (5)—CT may lead to psychosis through a path of heightened emotional distress (e.g., uncontrollable worry, sad mood, irritability, and fatigue). Previous research has proposed that traumatic events may cause structural and neurochemical abnormalities in the brain and nervous system, affecting the function of the immune-inflammatory system, the autonomic nervous system (ANS), and the hypothalamus-pituitary-adrenal axis (HPA-axis), which is involved in the stress response (41–44). This may result in an increased risk of uncontrollable worry and irritability. Moreover, according to the cognitive models of psychosis perspective, trauma may lead to diminished neuropsychological performance within domains of attention and cognitive flexibility (45). Even lead to negative beliefs about the self, world, and others (46). These beliefs may, in turn, lead to distressing interpretations of everyday events, eventually resulting in feeling down, depressed or hopeless or tired, or having little energy.

Results showed significant differences in total scores of depression, anxiety, and PTSD, as well as network characteristic among three groups (EA, PA, and SA). College students exposed to SA in childhood seem to have stronger connections between PTSD symptoms, such as sleep disturbance, difficulty concentrating, flashbacks, and emotional cue reactivity. Children can be sexually assaulted by adults and other kids who, because of their age or developmental stage, have authority over the victim or are in a position of trust or obligation for them. This result could be supported by previous studies (39, 40, 47). A study reported that psychiatric outpatients with a history of childhood SA had significantly increased rates of PTSD (40). Another study found that 86% of childhood SA survivors (*n* = 117) met DSM-III-R criteria for a PTSD diagnosis at some point during their lives. The duration and severity of the abuse were directly correlated with the severity and variance in PTSD symptoms (39). Individuals exposed to SA in childhood are more likely to have sudden intrusive memories of the original traumatic experience and a wave of the remembered emotional response (47). Previous studies reported that in survivors of sexual assault, there is a dysregulation of the HPA axis, which may be a primary source of the structural and functional abnormalities that contribute to PTSD symptoms (48). Through victim-blaming attitudes and the perpetuating of rape myths, sociological effects of attack promote the development of PTSD (42, 49, 50). For the network research, Yang et al. also reported a strong connection between sleep disturbance and difficulty concentrating (24), which might be explained by sleep disturbance arousing a series of adverse outcomes including lack of focusing (51, 52). The stronger associations between flashbacks and emotional cue-reactivity were also found in these studies (19, 21, 24). Understanding the associations between psychological symptoms affected by SA can potentially inform targeted efforts to prevent psychopathology.

Compared with PA and SA groups, the EA group is more likely to present stronger connections with suicide-related symptoms. A meta-analysis of longitudinal studies found that EA (OR = 3.98) was associated with suicide attempts (53). Another population-based review also reported that childhood EA was significantly associated with suicide-related behaviors (54). According to cognitive theories of psychosis, EA may result in negative thoughts about oneself, the outside environment, and other people. These assumptions might result in upsetting interpretations of commonplace occurrences (46). The shared social and biological surroundings between the child and the perpetrator are possible causal processes driving the EA and suicide-related symptoms (55).

Furthermore, any acquired brain damage resulting from EA in childhood might contribute to suicide-related behaviors (56). Shared biology might play a role in the link. For example, frightening, controlling, or isolating a possibly hereditary feature linked to suicide-related behaviors may be passed on biologically from parents to children, raising the risk of suicide-related behaviors in the offspring (57). Our results were in line with these results, which support that the EA group is more likely to present symptoms associated with suicide-related symptoms. Furthermore, a systematic review and meta-analysis from 124 studies suggested that EA individuals had a higher risk of developing depressive disorders (7). Therefore, it might be found that the EA group is more likely to present depressive symptoms like anhedonia, sad mood, and restlessness.

The PA group has shown stronger connections between anxiety symptoms (i.e., trouble relaxing, restlessness, and irritability). This result indicated that these associations were more critical in the affective symptoms network structure among college students exposed to PA in childhood. A systematic review reported that exposure to PA was associated with anxiety symptoms (OR = 1.7) (58). Moreover, individuals with a history of PA recognize anger affect more quickly and accurately than those without PA history (59). Perpetrators (i.e., parents or other family members) may legitimize PA in childhood as disciplinary in a social setting. The child may absorb the viewpoint and model it with anxiety-related symptoms as a sort of self-punishment (60).

Although this is a large-scale study containing the whole colleges in Jilin province, China, several limitations should be considered. First, the CTQ-SF is a self-report, retrospective measure of CT and may thus be prone to bias (e.g., social desirability, memory bias, and demand characteristics). Second, due to the cross-sectional design, the causation between psychosis symptoms cannot be confirmed. Third, some participants who suffered from depressive or anxiety disorders may be included in this study, which may bias the findings to an uncertain extent. Finally, we only compared a single form of CT group without consideration of overlap with other types, and further studies should be explored among overlapping forms of CT groups.

In conclusion, this network analysis revealed that uncontrollable worry, sad mood, irritability, and fatigue were the most core symptoms of psychotic symptoms among college students exposed to childhood EA, PA, and SA. Moreover, there are significant differences between the three groups. The SA group is more likely to be associated with PTSD symptoms, the EA group is more likely to be associated with suicide-related symptoms, and the PA group is more likely to be associated with anxiety symptoms. Therefore, it is recommended that schools and local communities should support early intervention to improve psychological wellbeing.

## Data availability statement

The raw data supporting the conclusions of this article will be made available by the corresponding authors, under reasonable request.

## Ethics statement

The study involving human participants was reviewed and approved by the Ethics Committee at Jilin University. The participants provided their electronic informed consent to participate in this study.

## Author contributions

Study design: YW, SX, and YJ. Study implementation and data collection: XS, XW, and HL. Data analysis: YJ and ZH. Manuscript: YJ, YW, SX, and JL. All authors read and approved the final manuscript.
