# Supplementary material for: Co-occurrence of PTSD and affective symptoms in a large sample with childhood trauma subtypes: A network analysis
Source: Front Public Health. 2023 Mar 7;11:1093687. doi: 10.3389/fpubh.2023.1093687 (PMC10028141; doi:10.3389/fpubh.2023.1093687)
Supplement: Supplementary file 1 [file Data_Sheet_1.docx]

**Supplementary Material**

Supplementary Table 1. Questionnaires and items used for the assessment of depressive, anxiety and PTSD Symptoms.

Supplementary Figure 1. Centrality indices of each node in the networks among three groups (EA, PA and SA).

Supplementary Figure 2. Bootstrapped confidence intervals of edge weights among three groups.

Supplementary Figure 3. Estimation of edge weight difference by bootstrapped difference test among three groups.

Supplementary Figure 4. Estimation of node strength difference by bootstrapped difference test.

Supplementary Figure 5. Stability of centrality indices by case dropping subset bootstrap.

Supplementary Figure 6. Comparison of network properties between emotional abuse and physical abuse participants.

Supplementary Figure 7. Comparison of network properties between emotional abuse and sexual abuse participants.

Supplementary Figure 8. Comparison of network properties between physical abuse and sexual abuse participants.

Supplementary Table 1. Questionnaires and items used for the assessment of depressive, anxiety and PTSD Symptoms.

|  | Reference Name | Item |
| --- | --- | --- |
| PHQ-9 | Anhedonia | Little interest or pleasure in doing things |
|  | Sad Mood | Feeling down, depressed or hopeless |
|  | Sleep | Trouble falling asleep, staying asleep, or sleeping too much |
|  | Fatigue | Feeling tired or having little energy |
|  | Appetite | Poor appetite or overeating |
|  | Guilt | Feeling bad about yourself – or that you're a failure or have let yourself or your family down |
|  | Concentration | Trouble concentrating on things, such as reading the newspaper or watching television |
|  | Motor | Moving or speaking so slowly that other people could have noticed；or, the opposite – being so fidgety or restless that you have been moving around a lot more than usual |
|  | Suicide | Thoughts that you would be better off dead or of hurting yourself in some way |
| GAD-7 | Nervousness | Feeling nervous, anxious or on edge |
|  | Uncontrollable Worry | Not being able to stop or control worrying |
|  | Excessive Worry | Worrying too much about different things |
|  | Trouble relaxing | Trouble relaxing |
|  | Restlessness | Being so restless that it is hard to sit still |
|  | Irritability | Becoming easily annoyed or irritable |
|  | Felling afraid | Feeling afraid as if something awful might happen |
| TSQ-10 | Intrusive thoughts | Upsetting thoughts or memories about the event that have come into your mind against your will |
|  | Nightmares | Upsetting dreams about the event |
|  | Flashbacks | Acting or feeling as though the event were happening again |
|  | Emotional cue reactivity | Feeling upset by reminders of the event |
|  | Physiological cue reactivity | Bodily reactions (such as fast heartbeat, stomach churning) |
|  | Sleep disturbance | Difficulty falling or staying asleep |
|  | Irritability/anger | Irritability or outbursts of anger |
|  | Difficulty concentrating | Difficulty concentrating |
|  | Hypervigilance | Heightened awareness of potential dangers to yourself and others |
|  | Exaggerated startle response | Feeling jumpy or being startled by something unexpected |

**Supplementary Figure 1. Centrality indices of each node in the networks among three groups (EA, PA and SA).**


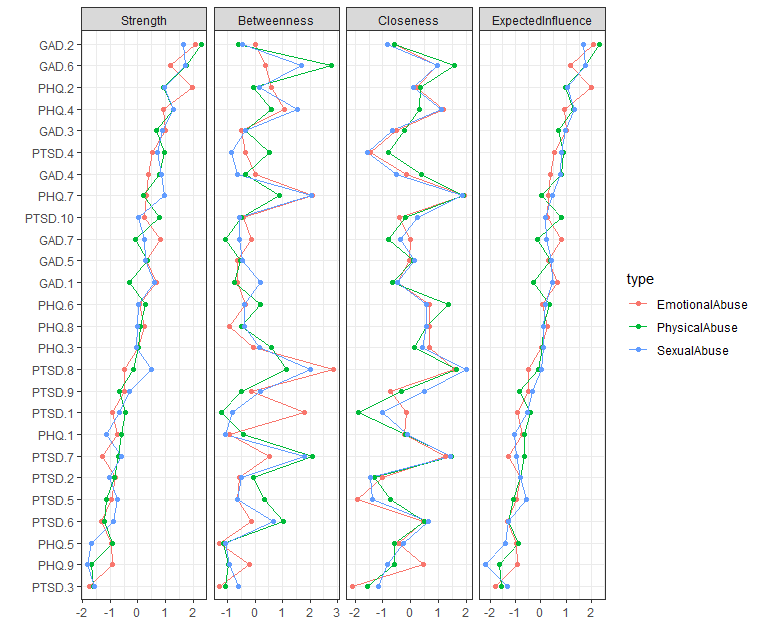


**Supplementary Figure 2. Bootstrapped confidence intervals of edge weights among three groups.**


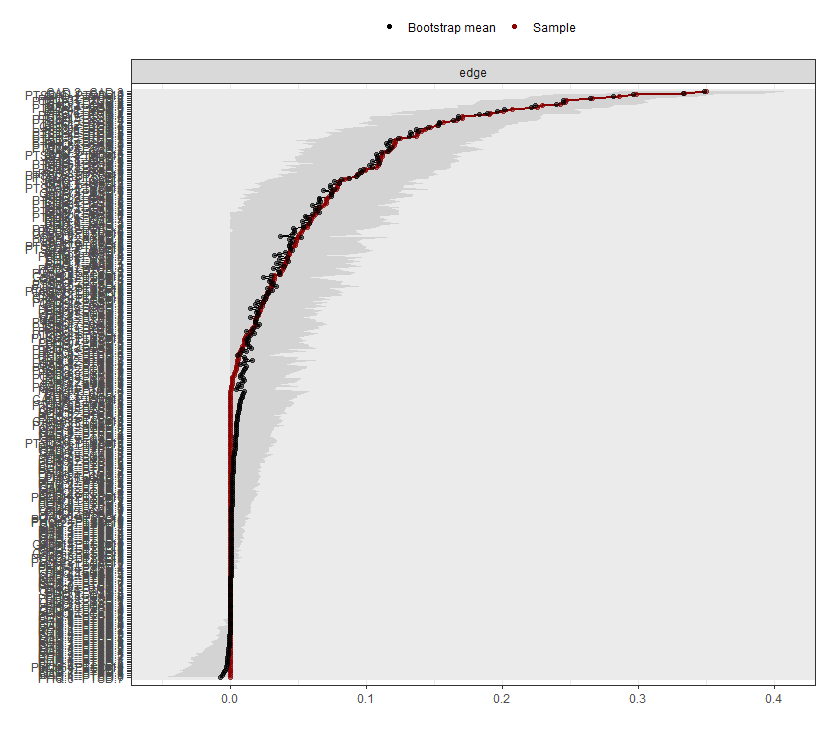

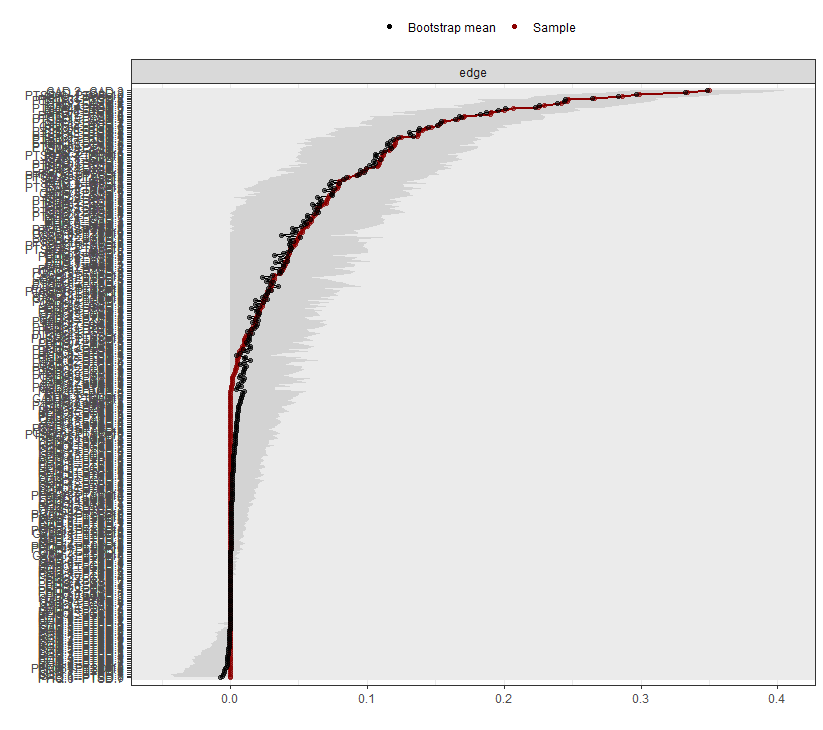

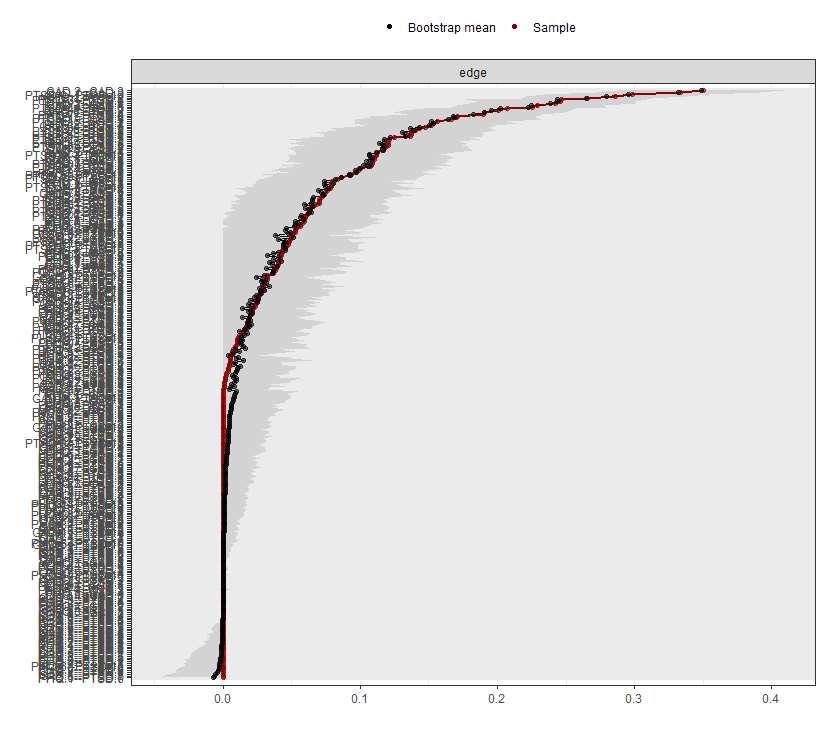


Left panel: EA group

Middle panel: PA group

Right panel: SA group

**Supplementary Figure 3. Estimation of edge weight difference by bootstrapped difference test among three groups.**


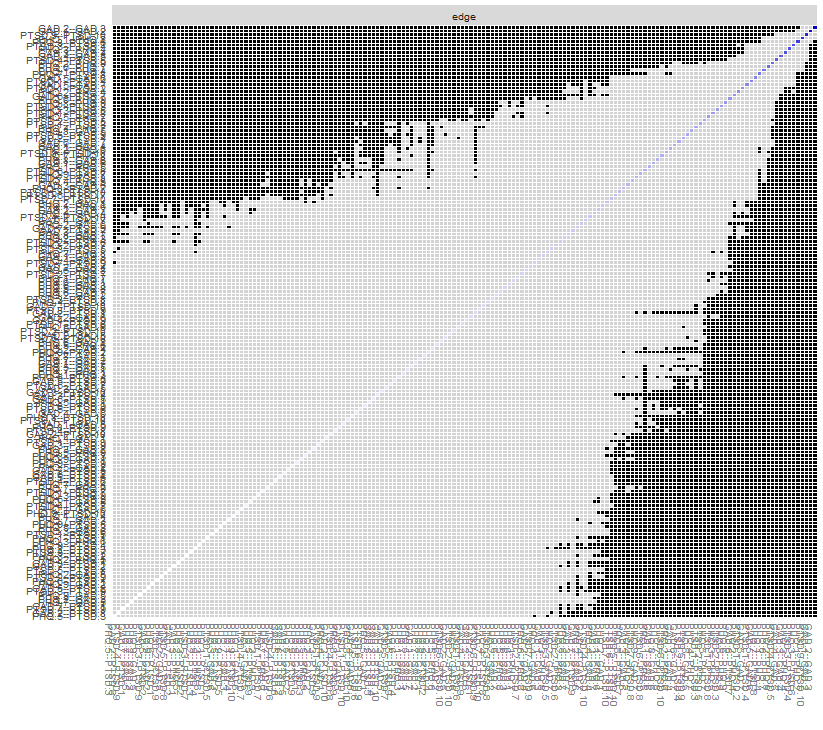

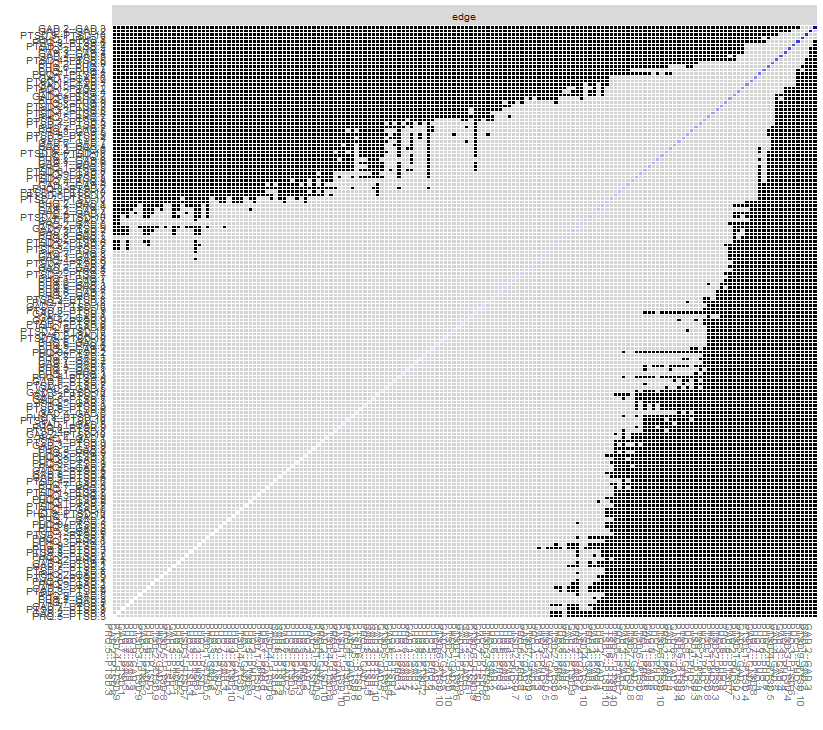

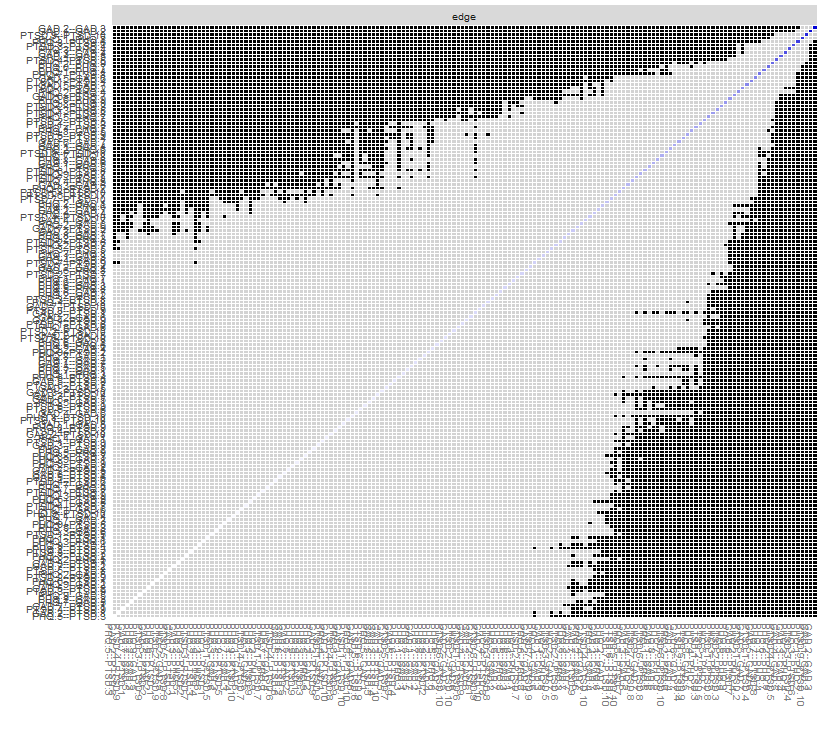


Left panel: EA group

Middle panel: PA group

Right panel: SA group

**Supplementary Figure 4. Estimation of node strength difference by bootstrapped difference test.**


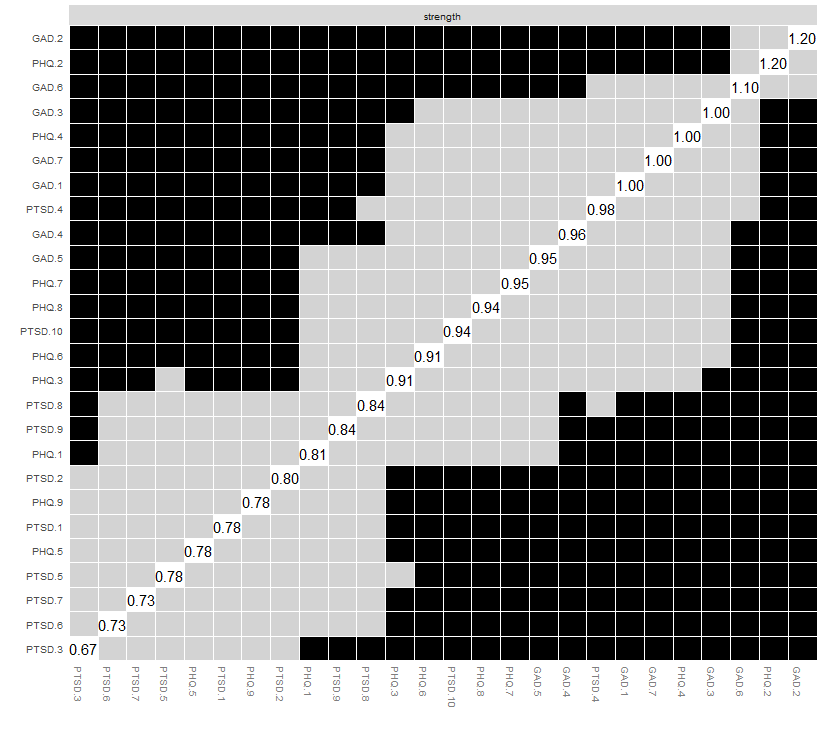

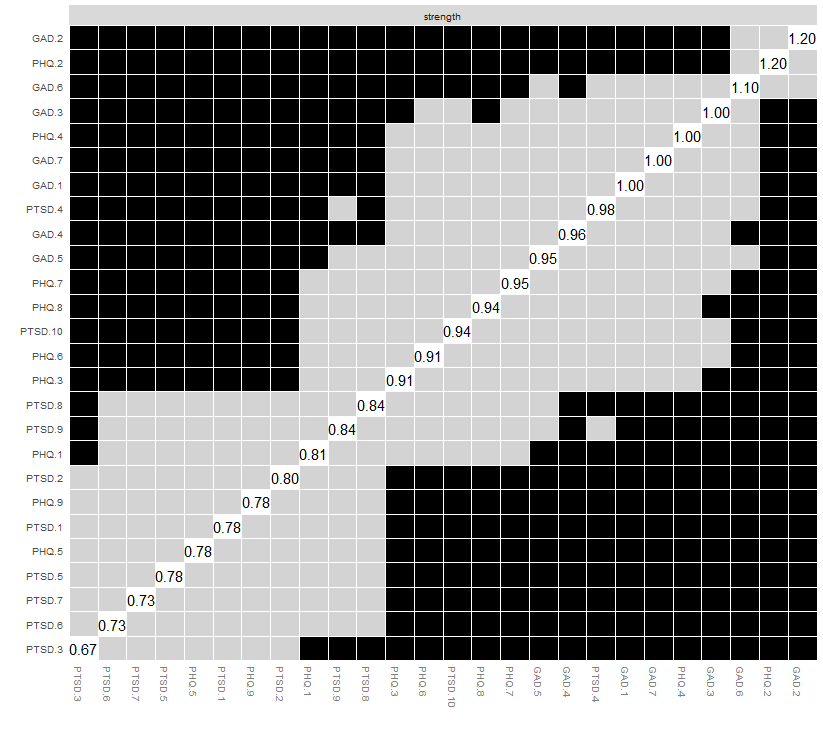

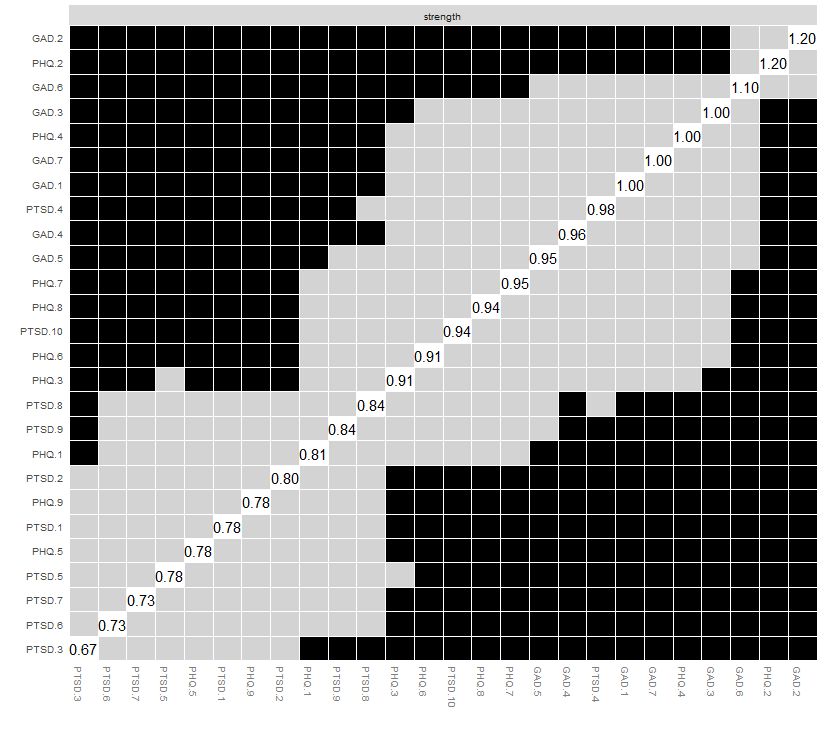


Left panel: EA group

Middle panel: PA group

Right panel: SA group

**Supplementary Figure 5. Stability of centrality indices by case dropping subset bootstrap.**


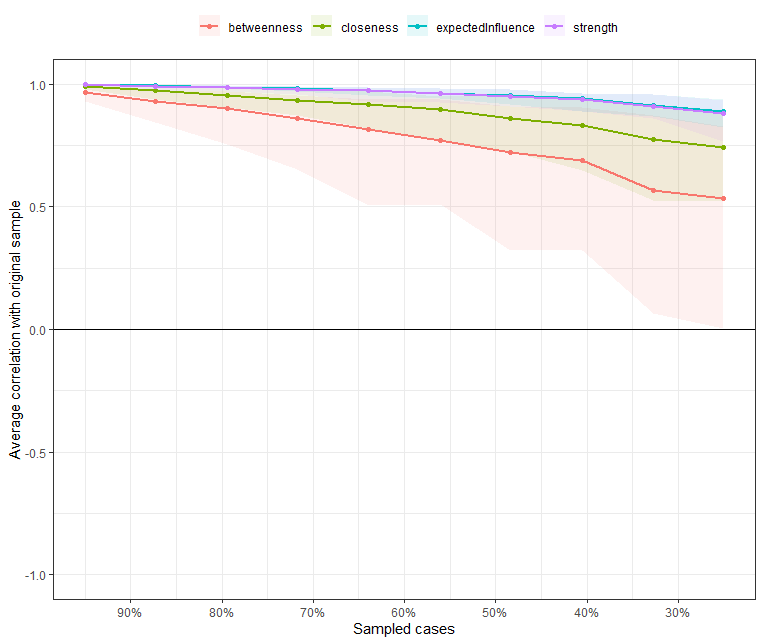

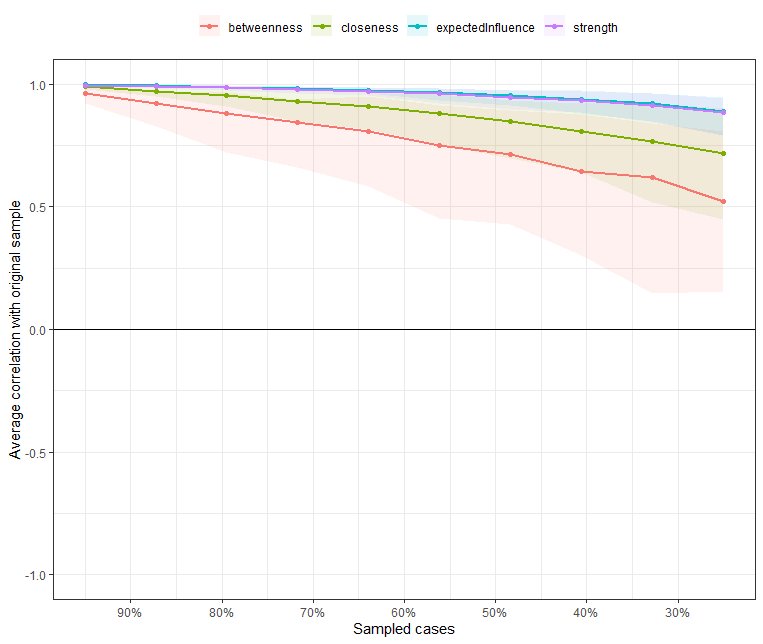

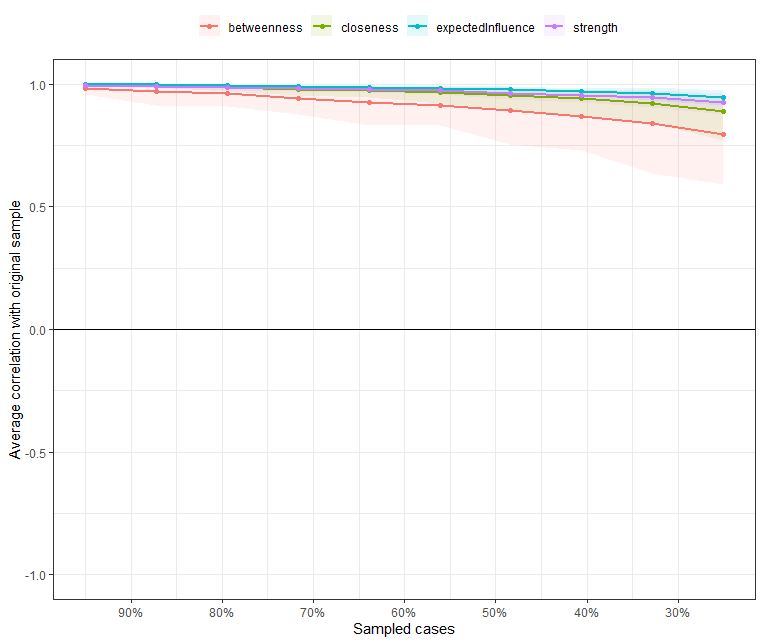


Left panel: EA group

Middle panel: PA group

Right panel: SA group


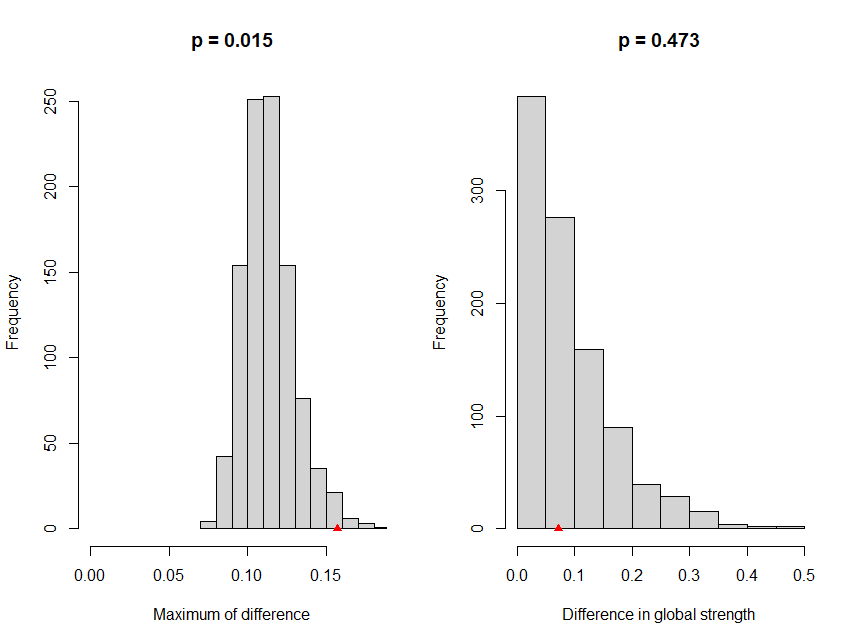


**Supplementary Figure 6. Comparison of network properties between emotional abuse and physical abuse participants.**

The Network Comparison Test (NCT) is a permutation test to investigate invariance in different network characteristics.

Left Panel: Plot of bootstrap value of the maximum difference in any of the edge weights (1000 permutations), with significant difference (M=0.16, p=0.015).

Right Panel: Plot of bootstrap value of the difference in network global strength, with no significant difference (network strength among emotional abuse participants: 11.79; among emotional abuse participants: 11.86; S: 0.07, p=0.473).

Invariance in edges weights was examined using the permutation test, generating sets of p values for each edge-edge comparison. Holm-Bonferroni corrected p values were all >0.05 indicating absence of significant differences.

**
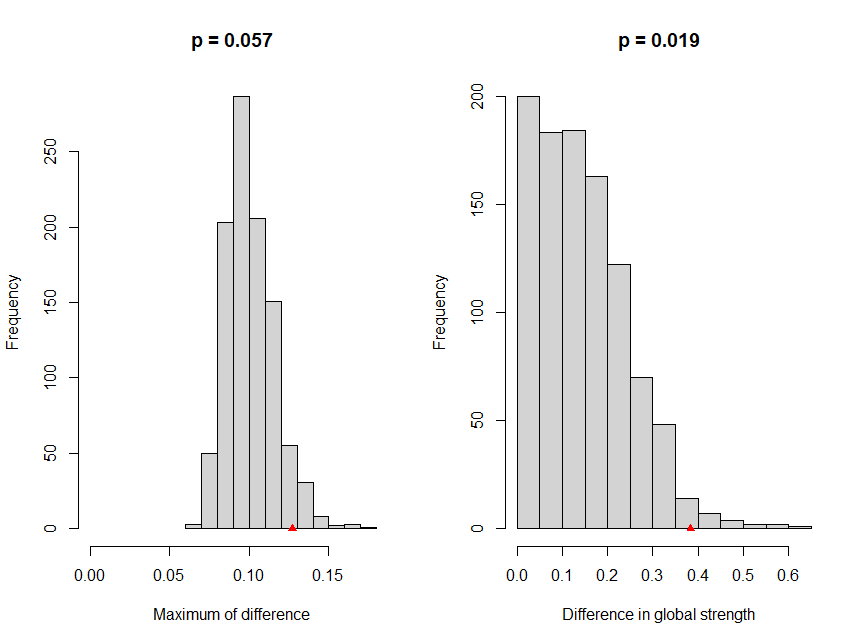
**

**Supplementary Figure 7. Comparison of network properties between emotional abuse and** **sexual abuse participants.**

The Network Comparison Test (NCT) is a permutation test to investigate invariance in different network characteristics.

Left Panel: Plot of bootstrap value of the maximum difference in any of the edge weights (1000 permutations), with no significant difference (M=0.12, p=0.057).

Right Panel: Plot of bootstrap value of the difference in network global strength, with significant difference (network strength among emotional abuse participants: 11.79; among sexual abuse participants: 12.18; S: 0.38, p=0.019).

Invariance in edges weights was examined using the permutation test, generating sets of p values for each edge-edge comparison. Holm-Bonferroni corrected p values were all >0.05 indicating absence of significant differences.

**
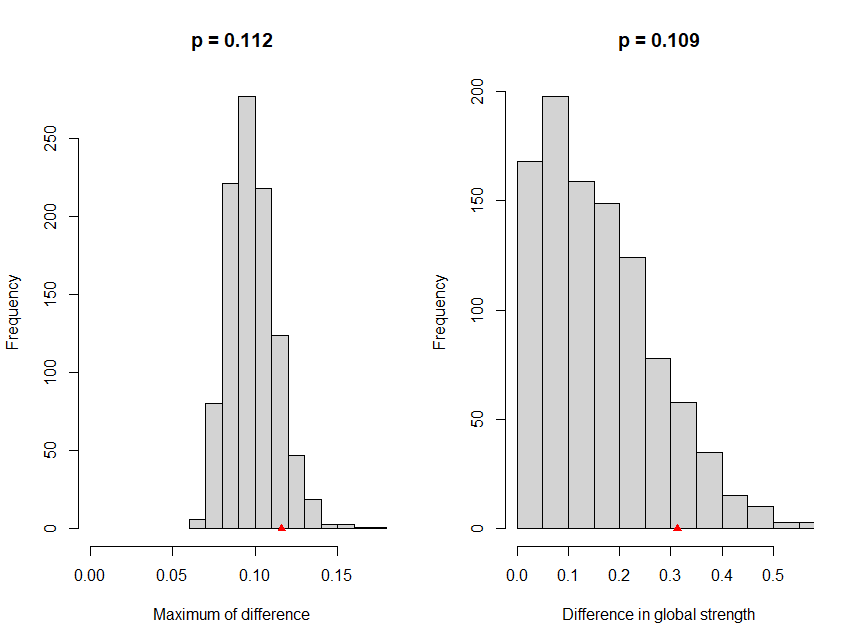
**

**Supplementary Figure 8. Comparison of network properties between** **physical abuse and sexual abuse participants.**

The Network Comparison Test (NCT) is a permutation test to investigate invariance in different network characteristics.

Left Panel: Plot of bootstrap value of the maximum difference in any of the edge weights (1000 permutations), with no significant difference (M=0.12, p=0.112).

Right Panel: Plot of bootstrap value of the difference in network global strength, with no significant difference (network strength among physical abuse participants: 11.86; among sexual abuse participants: 12.18; S: 0.31, p=0.109).

Invariance in edges weights was examined using the permutation test, generating sets of p values for each edge-edge comparison. Holm-Bonferroni corrected p values were all >0.05 indicating absence of significant differences.
